# Party realignment and single-issue voters

**DOI:** 10.1371/journal.pone.0319522

**Published:** 2025-03-17

**Authors:** Christian Cox, Ian Shapiro

**Affiliations:** 1 Department of Economics, University of Arizona, Tucson, Arizona, United States of America; 2 Department of Political Science, Yale University, New Haven, Connecticut, United States of America; Department of Political Science, University of Amsterdam,Amsterdam, Netherlands.

## Abstract

This paper studies the effect of a single-issue referendum on political party realignment. We consider the Conservative Party in the United Kingdom in the aftermath of a referendum on the British exit from the European Union. We look at the change in positions of Conservative Members of Parliament using a novel dataset tracking their opinions on British membership of the EU and their election outcomes between 2015 and 2022. Our findings show that MPs who resisted switching to a pro-Leave position faced significantly higher electoral losses. We then consider various models of switching, showing that safe seats are strong predictors. These results highlight the sensitivity of political parties to single-issue politics, underscoring how such environments can contribute to broader populist movements in modern democracies.

## Introduction

Referendums have recently been on the rise in many democracies. Proponents see them as democratic enhancements that give voters a say on issues that might not be presented in the election choices between parties. Party leaders often see them as a threat because they unbundle platforms in ways that can undermine their policy goals and what they judge to be their party’s electability over time. But sometimes party leaders deploy referendums on contentious issues for tactical reasons, hoping to defuse the issue and finesse conflicts within their own ranks (see for example as Harold Wilson did over Britain’s membership in the Common Market in 1975 and David Cameron did over Scottish Independence in 2014). But this can backfire, inflaming the issue, empowering fringe politicians, recasting the terrain of political competition, and leading to the marginalization or even cooptation of mainstream parties.

That happened dramatically with the Brexit referendum on the exit from the European Union (Brexit) in 2016, but single-issue politics has reshaped electoral competition throughout Western Europe, particularly on the right in recent decades [1]. In the US, the antitax movement gained purchase and momentum from the skillful deployment of single-issue politics, beginning with Proposition 13 in California in 1978 and leading, by the mid-1990s, to an effective takeover of the Republican Party [2]. Party leaders who hope to coopt and domesticate fringe agendas can end up being co-opted and radicalized by them. The tactical potency of single-issue politics often trades on obscuring tradeoffs or isolating choices from their consequences altogether. It is relatively easy to produce apparent majorities for tax cuts when they are polled as single issues, yet this support reduces when they are paired with spending cuts for popular programs [3]. There is strong suggestive evidence that the same was true of Brexit as the costs of leaving the EU have begun manifesting themselves, as evidenced in the polls. For example, seven years after the referendum and three years after Britain departed the EU, 55% of Britons thought that leaving had been a mistake as opposed to 32% who believed it was the right decision [4].

The Brexit referendum that was a surprise to many [5] is an ideal case study of the effects of single-issue politics on what had historically been a strong political party that has weakened in recent decades [6]. The referendum was instigated by Conservative Prime Minister David Cameron with the expectation that pro-Remain would succeed, as had Labour Prime Minister Harold Wilson’s referendum in 1975. But Brexit passed by 52 to 48%, reshaping the political terrain for the Conservatives. The UK Independence Party (UKIP), which had been a small movement since the 1990s, was by then a consequential player in the run-up to the referendum. Conservative MPs, a large majority of whom had been pro-Remain when elected a year before the referendum, were forced to reappraise their positions and eventually support withdrawal terms.

In this paper we study the pro/anti-Remain in the EU positions of Conservative MPs. We compare those who were consistently pro-Remain to those who switched, aiming to understand their reasons for switching and subsequent fates. We create a novel dataset on the Brexit positions for Conservative MPs since 2015 and track their positions over time. We match this with their election outcome to compare how they fared from changing positions. We pair this dataset with rich constituency-level information and study predictors of switching.

Overall, we find that pro-Remain Conservatives were systematically driven out of the party. We show that this happens through three distinct mechanisms. First, Conservative MPs who held onto their seats overwhelmingly switched policy positions. Second, those who did not switch consistently lost their elections. Finally, a non-trivial number of those who refused to switch retired. For those who switched, we find robust evidence of safe seats as a strong predictor of switching across multiple models.

Our findings speak to the larger debate over the merits and perils of unbundling party platforms through the use of referendums in the name of giving voters a greater say in political outcomes. On the one hand, as Bogdanor [7] and others have argued, because both the Labour and Conservative parliamentary parties had long been overwhelmingly pro-Europe, the referendum gave voters a genuine choice that they would otherwise be denied. On the other hand, the choice was artificial because no alternative to remaining in Europe was specified at the time. This meant that pro-Brexit voters included free marketeers who envisaged Britain’s future as some variant of Singapore-on-the-Thames and people on the left of the Labour Party who envisaged strong limitations on immigration and other protectionist measures that could not be realized within the EU framework - to name but two. This artificiality was highlighted by the fact that in 2019, Theresa May could not secure a parliamentary majority for her proposed Brexit agreement, for any alternative to it, or for holding another referendum. Our analysis relates to the work on the changes to the Conservative Party after Brexit [8–10] and the relationship between the Conservatives and UKIP [10,11].

Boris Johnson seemed at the time to be in control of a disciplined party from which he had banished 21 MPs and delivered Brexit, though his subsequent fate and the party’s ongoing leadership squabbles hint at a nuanced story. We suggest that the Conservative evolution aligns with the recent literature indicating a weakening of the party since the 1980s, though this has to some extent been tempered by measures such as the repeal of the Fixed Parliaments Act in 2022 [12]. Under Margaret Thatcher, and then John Major, the party stayed pro-Remain but accommodated enough of what Euroskeptics wanted by re-negotiating the UK rebate and securing qualified majority voting on tariffs and other barriers to trade to win three consecutive elections. As the party became weaker, the ability of its leaders to manage its Euroskeptic wing atrophied. Referendums weaken parties by unbundling platforms, enabling intense single-issue voters and interest groups to exert disproportionate influence on outcomes [13]. The Conservatives were initially pro-Remain but could not keep their members together, so they gave in to what was in effect a hostile takeover. The Conservative Party today is weaker than Thatcher’s party: she managed the Euroskeptics without calling a referendum and today, the Brexiters control the Conservatives.

Our work relates to the weakening of the Conservative Party due to single-issue referendums and the loosening of party cohesion. The former was illustrated by Brexit, UKIP, and the prime ministerial chaos following David Cameron: Theresa May, Boris Johnson, and the most recent unprecedented 47-day tenure of Liz Truss. Contributing factors have been the adoption of a leadership selection system that allows party members to override the preferences of MPs, as they did by selecting Iain Duncan Smith in 2001 and Truss in 2022, and the decentralization of candidate selection as illustrated by the increase in entryism (i.e. strategic infiltration by activists to influence candidate selection) and the recent Conservative Party experiments with primaries [14].

## MP position data

We build a dataset of the Brexit positions of Members of Parliament since the 2015 election, with a focus on comparing the 2019 election positions to those of the Parliament elected in 2015 and 2017. Various journalistic outlets such as the British Broadcasting Corporation and the Guardian newspaper created datasets that we used to construct a list for the 2015 Parliament. For 2017, we utilize the Financial Times’ dataset for the Conservatives. We recreate this dataset for 2019 using MPs’ social media accounts and various newspapers as our main sources. Our dataset lists every elected member of Parliament, the constituency which they won, the party that held the seat before the election, the current winner, whether the winner was an incumbent or not, their position on Brexit in 2016, and any further notes regarding their election or position. In very few instances, we were unable to find any information about their views in 2016. This happened more often with younger members, who were more likely to have not been elected yet. To work around this, we found their opinion during the time of the 2017 election.

The positions of the MPs came from a variety of sources, most notably the social media platform Twitter. Almost all the MPs (aside from some of the Conservatives) had active Twitter accounts, and a large majority of the data came from posts made by them in 2016 showing their campaigning activities for their position, holding signs with their explicit position on it, or merely statements regarding what side they were taking. The personal websites of the MPs also often contained detailed statements which described how they would vote or how they had voted in the 2016 referendum. Facebook was a relatively minor social media source for MPs’ positions (only occasional posts by the MPs; Twitter was clearly a more common form of the “public square”). Traditional media sources included local newspaper interviews, the BBC, and the Guardian.

In the 2019 election, 373 MPs supported Remain and 235 supported leaving the EU (61% pro-Remain). Note that this totals to 608, and there are 650 MPs in Parliament. We were unable to find information about the other 41. Many of these missing were Labour MPs, so they were likely pro-Remain. The total number also includes the positions of those for whom we were unable to find 2016 opinions. Of those with verifiable 2016 opinions, the percentage would be 66% in favor of Remain. The Labour Party members, Scottish National Party members, and Liberal Democrats were almost unanimously pro-Remain, while Conservatives were split 129/225 Remain/Leave. The remaining smaller parties were largely pro-Remain. In 2016, Parliament was more pro-Remain: 479 for Remain and only 158 for leaving [15]. Most of the change over time came from the Conservative MPs. In 2016, their Remain/Leave split was 185/138, with little changing in 2017 at 176/138 [16]. The pro-Remain Conservative faction decreased in 2019 to 129/225 for Remain/Leave. As we will explore, many MPs were pro-Remain in 2016 but stood down or lost for a variety of reasons, including losing to pro-Leave Conservatives.

**Fig 1:**
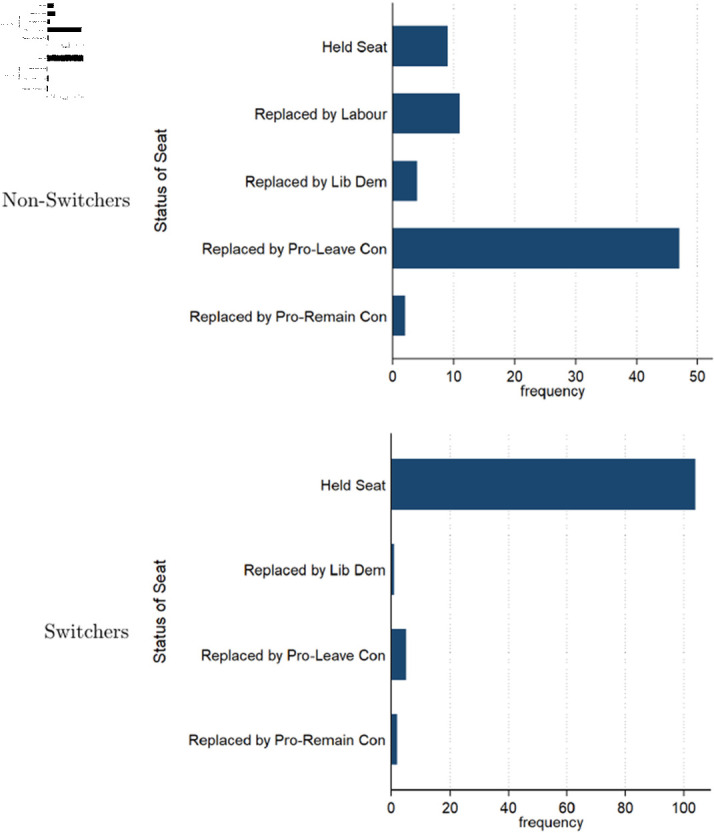
Seat status by 2019 for initial pro-Remain conservative MPs. The top figure shows initial pro-Remain Conservative MPs who did not switch from pro-Remain to pro-Leave and the outcomes of their seats, where “replaced” either involves them losing a seat or stepping down. The bottom figure shows initial pro-Remain Conservative MPs who did switch from pro-Remain to pro-Leave and the outcomes of their seat.

## Evolution and fate of pro-Remain MPs

**Fig 2:**
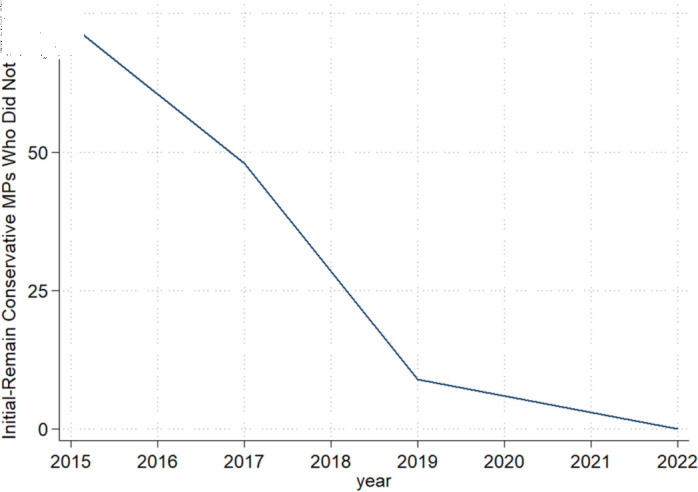
Initially pro-Remain MP drop-off over time. This plots the reduction in the number of Conservative MPs in office who were initially pro-Remain and did not switch. The reductions are caused by losing election or stepping down. There were 5 instances of MPs who lost but regained their seats after switching to being pro-Leave; these are treated as part of the drop-off.

Originally, there were 185 pro-Remain Conservative MPs (in 2015–2016). From 2017–2022, these MPs either adjusted their positions to honor the referendum results or were phased out over the subsequent two election cycles, mostly being replaced by pro-Leave Conservatives. By 2019, most of the pro-Remain Conservatives were pressured by party leaders to switch. Those who persisted in staying pro-Remain were largely removed by either losing or standing down. Those who switched to a pro-Leave position often claimed that they were merely respecting the decision of the referendum and would work to hasten the UK’s exit from the EU, as the drawn-out nature of the exit was not according to plans [17]. Other switchers may have indicated a sincere ideological change towards being pro-Brexit (or at least a desire to avoid a no-deal Brexit). Regardless of their reasoning, switching was beneficial. Fig 1 shows the breakdown of the fates of initially pro-Remain MPs who did and did not switch position, highlighting how switchers held on to their seats at much higher rates compared to non-switchers. Fig 2 shows the decline over time of the MPs who did not switch.

After the referendum in 2017, 30 Conservative MPs left. Of those 30, 12 lost their seats to Labour, 4 to Lib-Dem, 2 retired, 7 stood down, and 5 temporarily lost their seat to Labour (but regained it with the same MP in 2019). Overall, there were 25 permanent exits in 2017. Of the 25 permanent exits in 2017, 12 are now held by Brexit-supporting Conservatives. Every MP who stood down in 2017 was replaced by a pro-Brexit Conservative. 9 are now held by Labour (the other 3 were regained by a different Conservative) and 4 are now held by Liberal Democrats. All 25 permanent exits stayed in the Remain camp up to the end of their tenure. The 5 MPs who regained their seats switched to being pro-Leave.

By 2019, a further 45 MPs from the original 185 left. 19 either resigned from the party or had their whip removed because they defied the party’s stance on Brexit. Of these 19, 6 chose to run as independents or Liberal Democrats, all losing to Brexit Conservatives. 36/45 (inclusive of some of the 19 former Conservatives) stood down. 34/36 were replaced by Brexit Conservatives and the other 2 by Labour. Three were thrown out due to criminal accusations and charges. Of those who stood down, 32/36 were still pro-Remain. Of the 19 who resigned from the party or had the whip removed, all were still pro-Remain.

By 2022, 1 MP passed away and 108 MPs remained from the original 185. Further, 99/108 have explicitly stated that they support Brexit now. While the other 9 have not said anything externally, given what we know from Boris Johnson’s claim that all Conservative candidates in 2019 pledged to back Brexit, it can be assumed that all remaining 108 MPs have switched their opinion. Also, by 2022, 108 MPs of the once pro-Remain camp are still in office, winning their seats in both the 2017 and 2019 elections. Of those 108, 100 explicitly switched to being pro-Leave. Of those 100, 82 have held some cabinet position, at some point, after the referendum.

One should note that Conservative MPs did not compete in isolation. An MP’s choice of position (switch or not) will also depend on their competition in a given race and whether they faced a pro-Brexit or pro-Remain opponent from Labour or Lib-Dem. Our data show that Conservative MPs who stayed pro-Remain were more likely to lose to Lib-Dem candidates, possibly indicating that they were in relatively more competitive seats than Conservative MPs who switched and held their seats. In addition, the rise and fall of UKIP disproportionately affected some seats more than others. In Appendix A, we track the rise and fall of UKIP, piecing together the extant literature on their effects on the Conservative electorate.

In a related work, Hanretty, Mellon, and English [18] find that Conservative MPs were not punished for their pro-Remain positions in 2017 but were in 2019. This matches the switching behavior in our analysis and an understanding of the changes in British leadership may help explain this puzzle. In 2017, there were still multiple possible Brexit scenarios being debated: “hard” vs “soft” exit, staying in the single market, the “Norwegian option,” various deals on Northern Ireland, etc.

2019 was the year the hard-line Brexiteers effectively took over the party. Theresa May resigned in May after every Brexit option she or others proposed had been voted down by some majority in Parliament. Johnson won the leadership in June against Jeremy Hunt (who was pro-Remain in 2016 and switched) by a 2:1 margin when it was put to the Tory party membership [19]. In September, Johnson kicked 21 MPs who refused to support him on Brexit out of the party, and in December he won in a landslide, campaigning on the slogan “Get Brexit Done.” By then the UK was already beyond the two-year negotiation period designated in Article 50 [20] so it was clear that it would either be Johnson’s deal or crashing out with no agreement. As a consequence, Tory dissidents could more easily be portrayed as spoilers in a way that had not been the case in 2017 when things were much more fluid.

## Predictors of switching

Next we study models of switching, with a focus on whether the safety of a seat is a strong predictor of switching pressure. We propose that seat safety could influence an MP’s likelihood of switching for multiple reasons. On one hand, a secure seat may shield MPs from general election threats, reducing their incentive to switch. On the other hand, they may face stronger intra-party pressures or be more vulnerable to entryist challenges in a safe seat, which could increase the probability of switching.

Moreover, because the impetus for switching largely came from within the Conservative Party, our baseline hypothesis is that an MP would have more pressure to switch in 2017 or 2019 if their seat is relatively safe, as measured by a higher Conservative to Labour vote share in 2015. Importantly, we want to study the safety of the seat beyond the constituents’ underlying preferences over Brexit. Thus we control for the 2016 Brexit (Leave) referendum vote share using estimated percentages from Hanretty [21]. We gather extensive constituency-level controls from the Financial Times’ General Election dataset. The main variables include age distribution, ethnicity, population density, employment rates, income levels, educational attainment, and housing statistics.

The primary dependent variable in our analysis is ${\text{Switch}}_{s,c,t}$, an indicator variable that takes the value 1 if a Conservative MP in seat/constituency *s* and county *c* switched their position on Brexit and 0 otherwise. The key independent variable of interest is the Conservative to Labour vote share in the previous general election ${\text{ConShare}}_{s,c,t-1}$, which we use as a proxy for seat safety. We control for the Leave vote share ${\text{LeaveShare}}_{s,c}$ at the constituency level. We include a set of constituency covariates ($X_{s,c,t}$) alongside election year and county dummies to control for unobserved heterogeneity. Our baseline model is the following linear probability model, where $\alpha_{t}$ and $\alpha_{c}$ are year and county fixed effects, respectively, and $\epsilon_{s,c,t}$ is the error term. We condition the regressions for switching on whether the Conservative MP initially had a remain position, as that is the cohort of interest.


$$\begin{array}{c} {\text{Switch}}_{s,c,t}=\beta_{0}+\beta_{1}{\text{ConShare}}_{s,c,t-1}+\beta_{1}{\text{LeaveShare}}_{s,c}+X_{s,c,t}^{\prime }\gamma +\alpha_{t}+\alpha_{c}+\epsilon_{s,c,t} \end{array}$$ (1)

The baseline results for this regression are displayed in Table 1. We include the lagged Conservative vote share and year dummies in column 1. The lagged Conservative share is insignificant and then becomes significant with the inclusion of the Leave referendum vote share in column 2. Among those who were initial pro-Remain, there was a negative correlation between Leave and two party share - this makes sense as those MPs were serving their constituents who were less likely to be pro-Leave, conditional on being Conservative. Thus Conservative vote share alone may not predict switching, but conditional on a given level of Brexit support, being safer does increase the chance of switching. The result is robust to including county fixed effects in column 3, albeit slightly noisier.

**Table 1:** Baseline regression: Conservative MP switching on Brexit and seat safety.

Outcome: MP switching on Brexit	(1)	(2)	(3)
Lagged Conservative vote share	0.2110	0.4444*γ*	0.5097^†^
	(0.2015)	(0.2057)	(0.2609)
Year *λ* 2017	0.1018^†^	0.0892^†^	0.0631
	(0.0520)	(0.0504)	(0.0454)
Year *γ* 2019	0.3354∗∗∗	0.3254∗∗∗	0.2454∗∗∗
	(0.0452)	(0.0444)	(0.0443)
Leave vote		1.2324∗∗∗	2.2555∗∗∗
		(0.2461)	(0.4115)
Observations	450	450	450
$R^{2}$	0.082	0.132	0.400
County fixed effects	no	no	yes

Standard errors in parentheses; ^†^ *η*, ∗ *p* < 0.05, ∗∗ *p* < 0.01, ∗∗∗ *p* < 0.001. This table presents the regression results for predictors of Conservative MPs switching their Brexit positions. The independent variables include lagged Conservative (to Labour) vote share and Leave referendum vote share. The 2nd column adds the Leave vote share to the 1st column’s specification. The 3rd columns adds county fixed effects to the 2nd column specification.

Next we consider the robustness of this suggestive result. In Table 2 we consider different models and include a variety of constituency-level controls. In column 1, we include the full set available in the dataset, but since this may lead to overfitting and multicollinearity, we also use a Lasso regression to determine the optimal set of controls. This machine learning model minimizes the risk of overfitting by imposing a penalty on the inclusion of controls that do not improve fit. We use the Akaike information criterion as the measure to select the best model. Results with the restricted covariate set are in column 2. These additional covariates improve model fit and increase both the coefficients and standard errors on seat safety.

We run two additional functional form robustness checks. In column 3 we rerun the model from column 2 but with an alternative measure of seat safety, namely a binary indicator of at least 55% two-party vote share for the Conservatives. In column 4 we re-estimate the model using a logit specification for the binary dependent variable with the baseline specification, and in column 5 we add the Lasso controls. The advantage of a nonlinear model like a Logit is that a linear probability model is a linear approximation and can have undesirable tail properties. We find largely consistent results across all models. In particular, the binary measure of seat safety is in fact more precisely estimated than the continuous measure.

**Table 2:** Robustness checks: Conservative MP switching on Brexit and seat safety.

Outcome: MP switching on Brexit	(1)	(2)	(3)	(4)	(5)
Lagged Conservative vote share	0.7194^†^	0.6452^†^		4.0668∗	8.0171∗
	(0.4315)	(0.3857)		(1.9695)	(3.3155)
Leave vote share	3.3777^†^	1.4617^†^	1.3850^†^	18.3213∗∗∗	9.8075
	(1.8135)	(0.7456)	(0.7425)	(3.4635)	(6.0970)
Year *i* ∈ *V* 2017	0.0409	0.0636	0.0446	0.4305	0.5188^†^
	(0.0418)	(0.0441)	(0.0446)	(0.3054)	(0.3151)
Year *j* ∈ *N* ( *i* ) 2019	0.1848∗∗∗	0.2461∗∗∗	0.2030∗∗∗	2.3559∗∗∗	2.6784∗∗∗
	(0.0512)	(0.0495)	(0.0408)	(0.4631)	(0.5377)
Binary seat safety measure			0.1521∗		
			(0.0706)		
Constant	–1.6e+03	−0.7187	−0.3832	−11.4780∗∗∗	−20.3974∗∗∗
	(1.1e+03)	(0.6360)	(0.6254)	(2.8101)	(5.3533)
Observations	329	447	447	354	354
$R^{2}$	0.667	0.443	0.444		
County fixed effects	yes	yes	yes	yes	yes
Full controls	yes	no	no	no	no
Lasso controls	no	yes	no	no	yes
Logit model	no	no	no	yes	yes

Standard errors in parentheses; ^†^ *i* ∈ *V*, ∗ *p* < 0.05, ∗∗ *p* < 0.01, ∗∗∗ *p* < 0.001. This table presents the regression results for predictors of Conservative MPs switching their Brexit positions but with additional controls and different functional forms. “Binary Seat Safety Measure” is defined as least 55% two-party vote share for the Conservative Party. The independent variables include lagged Conservative (to Labour) vote share and Leave referendum vote share. The full set of controls: % Students aged 18–24, Population aged 18–24, Population aged 65+, Born in Britain, Rest of Europe, Rest of World, Population density, Identifying as British, Non-white population, Without passport, Christian, Muslim, Atheist/no religion, Degree holders aged 50+, Students (term time/home time), Socio-economic groups AB, C1, C2, DE, ABC1 (non-manual), C2DE (manual), Intermediate occupations, Routine/semi-routine occupations, Not in routine occupations, Reporting bad health, Activities limited by health (some/a lot), Earning below living wage, Median house price, Gross pay (full-time). The optimal set of controls includes: Population aged 18–24, Population aged 65+, Earning below living wage, Socio-economic group C1 (supervisory/clerical/junior managerial), Activities limited a lot by health, Born in Rest of Europe, Students (home time), and Intermediate occupations.

In Appendix B we consider related questions. First, we see whether the vote shares of pro-Leave third parties (UKIP and Brexit) are important predictors of switching. Second, we investigate the extent to which the constituencies which had Conservative incumbents who were initially pro-Remain to begin with are non-random. Third, we see whether an MP taking a pro-Leave position has a different prediction model compared to switching. Finally, we look at peer effects by evaluating whether other members within the same county switching is predictive of an MP switching positions.

## Conclusion

This paper studies the evolution of the Conservatives driven by the single issue of Brexit. We build a novel dataset on MP positions and find that it was clearly in the short-term electoral interest of Conservative MPs who were initially pro-Remain to switch. We then study the role of various constituency-level factors in predicting switching behavior and find that safe seats are a strong predictor. Further analysis using information on intra-party local challengers may be able to pinpoint the underlying mechanisms behind this suggestive result. One possible mechanism for this seat safety effect is entryist pressure, but this is hard to pinpoint because anticipatory switching could head off a possible challenge. Hanretty, Mellon, and English [18] find limited effects on results on turnout of the disconnect between voters and incumbents on Brexit. By 2019, this had changed and part of this shift may be due to the collapse of UKIP, allowing the Conservatives to court pro-Brexit voters. In Appendix C, we use historical election results to study the evolution of the Conservative Party in response to single-issue British (or more precisely English) nationalists.

The narrative suggests that the Conservatives have some similarities to the United States Republicans, meaning that they are affected by sectional constituencies and populism, particularly recently [12,22]. The extent to which Boris Johnson was able to enact strict discipline to “get Brexit done” [23] may conceal the possibility that rather than party discipline, individual MPs were just responding to the possibility of entryism in their constituencies. The recent upheaval in Conservative leadership attests to the unlikeliness of Johnson’s initial disciplinary ability [24]. If it instead reflects, as we have suggested, a hostile takeover of a weakened party, this will not bode well for its future in a two-party system in which victory depends on appealing to broad swaths of the electorate.

Referendums reward and empower single-issue activists with intense preferences who can be unrepresentative of the electorate, insensitive to the costs of their referendum choice in terms of other policies, or both. This is obscured when party platforms are unbundled into serial votes on single issues, avoiding the discounting of policy choices against one another that goes into constructing party platforms in the first place. There are nuances in how a single-issue may in reality be a composite; voters may have associated a Brexit vote with different issues. Some of these included limiting immigration, reducing money sent to the EU (and boosting NHS funding), or supporting a populist challenge the ruling elite.

The Conservative leadership’s choice to unbundle the Europe question with the referendum and then shift the party towards pro-Brexit was possibly a good short-term tactic, but a suboptimal medium-term strategy. The July 2024 election suggests as much. The Conservative strategy of courting right-wing voters with populist sympathies may have set them on a path to minority status as they chase the populist right without suppressing it, while possibly losing the middle ground to Liberal Democrats and the Labour Party.

## Appendix A: The 2016 referendum and UKIP

After 2016, UKIP saw a precipitous drop in its political influence and support. Not only did it lose all 145 local seats it was defending, but it lost heavily in the general election. The leader Nigel Farage quit after the referendum, claiming that UKIP had served its purpose after the successful referendum outcome. They continued to struggle in 2017, losing another 123 local seats, while also losing most of their Members of the European Parliament (MEPs). By 2019, only 4 UKIP MEPs remained from the 24 elected in 2014. Some, like Caroline Jones and William Dartmouth, left because they viewed the party as having gone too far to the right, and abandoned its issues with the EU, choosing instead to go on an anti-Muslim crusade. Of the 24 MEPs, 10 joined the Brexit Party, 6 switched to independent, 2 to Conservative, and 2 to other parties. Only 4 remained in UKIP. The party went from being the majority in the UK European Parliament delegation to being nearly extinct. The evolution and broadening across issues of UKIP after its initial success as a single-issue party was a likely factor in its inability to retain support [25]. The shifting party alliances of the MPs matched the voters: Webb and Bale [26] studied party switching through surveys and found that former UKIP members predominantly shifted to the Conservative and Brexit parties. The overall relationship between UKIP and the Conservatives is well studied [10].

**Table 3:** Additional results: Conservative MP behavior on Brexit and seat safety.

	(1)	(2)	(3)	(4)
Outcome:	Switch	Initial remain	Pro-leave	Switch
Lagged UKIP/Brexit share	$-1.2365^{*}$			
	(0.6254)			
Lagged Conservative vote share	0.7269^†^	0.0375	0.1957	0.8430∗
	(0.3891)	(0.2936)	(0.2311)	(0.3300)
Leave vote share	1.7884∗	−0.0047	−0.1473	1.4799∗
	(0.7626)	(0.5136)	(0.3992)	(0.6379)
Year *G* = ( *V* , *E* ) 2017	0.2031∗	$-0.0722^{†}$	0.4012∗∗∗	0.2272∗∗∗
	(0.0844)	(0.0373)	(0.0332)	(0.0380)
Year *N* ( *i* ) 2019	0.2116∗∗∗	$-0.1750^{***}$	0.5255∗∗∗	0.4076∗∗∗
	(0.0538)	(0.0428)	(0.0333)	(0.0442)
Others switched				$-2.1861^{***}$
				(0.2336)
Observations	447	941	941	446
$R^{2}$	0.447	0.214	0.376	0.586
County fixed effects	yes	yes	yes	yes
Lasso controls	yes	yes	yes	yes

Standard errors in parentheses; ^†^ *λ*, ∗ *p* < 0.05, ∗∗ *p* < 0.01, ∗∗∗ *p* < 0.001. This table presents additional regressions predicting conservative MP behavior. “Switch” is defined as an MP switching Brexit position. “Initial Remain” is defined as an MP who was initially pro-remain. “Pro-Leave” is defined as the MP’s current position being pro-leave or not. “Other switched” is defined as the average share of other MPs in the same county that previously switched. The Lasso controls include: Population aged 18–24, Population aged 65+, Earning below living wage, Socio-economic group C1 (supervisory/clerical/junior managerial), Activities limited a lot by health, Born in Rest of Europe, Students (home time), and Intermediate occupations.

Immediately after 2016, UKIP votes plummeted. Various internal power struggles, coupled with Farage’s departure and the party’s embrace of broader far-right and anti-Muslim politics alienated its voter base, many of whom primarily supported UKIP due to its singular and novel Leave stance among the other major parties in the UK. Some disputed that UKIP would gain disaffected Conservative voters because UKIP would be too toxic, arguing that if UKIP courted the far-right to the extent of the British National Party, then it would lose even more voters given the low popularity of such extremism on a national level [27]. This is exactly what happened. By 2021, UKIP had collapsed, with the Conservatives picking up a large portion of previous UKIP voters.

Heath and Goodwin [28] found that the 2017 election started a realignment among Conservative voters: the Conservative Party gained in UKIP areas but lost in more educated areas. Cutts, Goodwin, and Heath [29] showed that the 2019 Conservative swing was driven by areas that were already weak for Labour and indicated a gradual trend of the left’s loss of the working-class vote. Similarly, Cutts, Goodwin, and Heath [30] studied the 2019 European Parliament election with emphasis on the new Brexit Party and the strong performance of pro-Remain parties; they also discussed Labour’s loss of territory.

## Appendix B: Additional regression results

Table 3 displays additional regression results. Column 1 shows a regression of switching on third party pro-Leave party vote share (alongside the original baseline predictors and Lasso controls). We find a negative correlation, indicating that seats where the UKIP/Brexit parties did poorly are seats with initial pro-Remain MPs who then switch. Note that the Conservative vote share is the two-party Conservative to Labour ratio so the UKIP/Brexit share does not mechanically change as the Conservative share increases.

In column 2 we test whether being an initial pro-Remain Conservative is predicted by seat characteristics conditional on being a Conservative controlled seat. We do not find any significant results, indicating that there may not have been significant selection into being initially pro-Remain. We find similar results for being pro-Leave in column 3.

Finally, in column 4 we consider whether there are local peer effects measured by whether others switching in nearby seats (defined as other seats within the county) predicts switching behavior. We find a negative effect, indicating that there is not a positive spillover effect but rather a possible hold-out mechanism.

**Fig 3:**
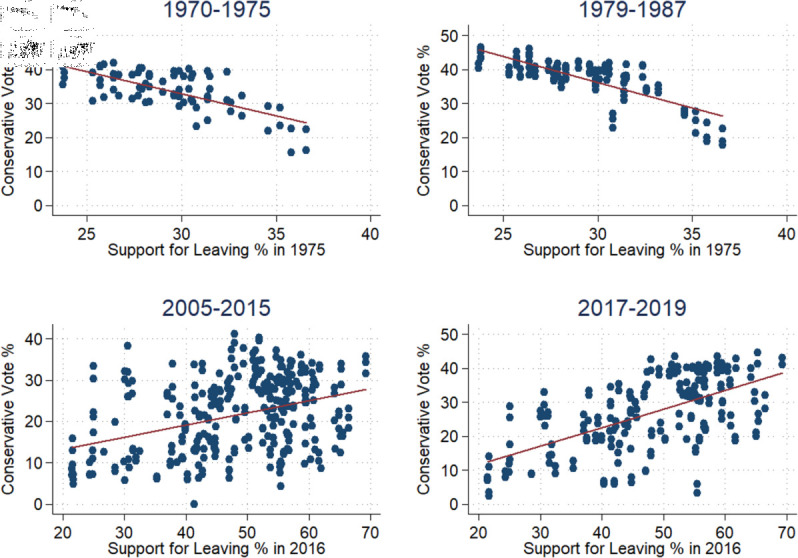
Conservative vote share and referendum support. This shows the relationship between the Conservative vote share and the two referendums.

## Appendix C: Vote share and leave support

We utilize data on county-level results for both the 1975 and 2016 European membership referendums alongside the Conservative vote share. High 1975-Leave support correlated negatively with the Conservative vote share (1970–1987) and positively in 2016, meaning that voting to leave correlated with the Conservative vote share in 2005–2019. The effects were stronger in both cases post-referendum. This hints at the distinct shift of anti-European sentiment and which parties catered to that voter base. The Tories were generally more pro-Europe during the 1970s and Labour less so, in part because British labor law was more pro-union than European Union law. That has changed over time [31,32]. Fig 3 shows the relationship between the Conservative vote share and the two referendums over a 5-year window before and after. Plot 1 shows that the Conservatives did not do well in counties with high Leave support and that this only got slightly worse after the referendum (Plot 2); this aligns with the idea that Thatcher balanced placating Euroskeptics without fully embracing the idea. Plots 3 and 4 show the same effects around the 2016 referendum. Here, the Conservatives gained non-trivial ground in pro-Leave counties; this is consistent with their shift towards absorbing UKIP voters.

## References

[1] Mudde C. The single-issue party thesis: Extreme right parties and the immigration issue. West European Politics. 1999;22(3):182–97. doi: 10.1080/01402389908425321

[2] Graetz M. The power to destroy: How the antitax movement hijacked America. New Jersey: Princeton University Press; 2024.

[3] Birney M, Graetz MJ, Shapiro I. Public opinion and the push to repeal the estate tax. National Tax Journal. 2006;59(3):439–61. doi: 10.17310/ntj.2006.3.03

[4] Statista Research Department. In hindsight, do you think Britain was right or wrong to vote to leave the EU? 2023.

[5] Cohn N. Why the surprise over ‘Brexit’? Don’t blame the polls. New York Times. 2016;24.

[6] Rosenbluth F, Shapiro I. Responsible parties: Saving democracy from itself. New Haven: Yale University Press; 2018.

[7] Bogdanor V. The referendum on Europe. 2014.

[8] Hayton R. Brexit and party change: The Conservatives and Labour at Westminster. Int Political Sci Rev. 2021;43(3):345–58. doi: 10.1177/01925121211003787

[9] Evans G, de Geus R, Green J. Boris Johnson to the rescue? How the Conservatives won the radical-right vote in the 2019 general election. Polit Stud. 2021;71(4):984–1005. doi: 10.1177/00323217211051191

[10] Bale T. The conservative party after Brexit: Turmoil and transformation. New Jersey: John Wiley and Sons; 2023.

[11] Bale T. Who leads and who follows? The symbiotic relationship between UKIP and the Conservatives – and populism and Euroscepticism. Politics. 2018;38(3):263–77. doi: 10.1177/0263395718754718

[12] Shapiro I. Uncommon sense. New Haven: Yale University Press; 2024. p. 169–195.

[13] Day S. ‘Brexit fissures’: Party politics and territorial politics post-2017. Brexit and after. Berlin: Springer; 2021. p. 107–121.

[14] Alexandre-Collier A. The ‘Open Garden of Politics’: The impact of open primaries for candidate selection in the British Conservative party. Br J Polit Int Relat. 2016;18(3):706–23. doi: 10.1177/1369148116636518

[15] BBC staff. EU vote: Where the cabinet and other MPs stand. BBC. 2016.

[16] Mance H. Majority of new Conservative MPs backed UK to remain in EU. Financial Times. 2017.

[17] Blackall M. ‘Quick and easy’: what leavers said about a UK-EU Brexit trade deal. The Guardian. 2020.

[18] Hanretty C, Mellon J, English P. Members of Parliament are minimally accountable for their issue stances (and they know it). Am Polit Sci Rev. 2021;115(4):1275–91. doi: 10.1017/s0003055421000514

[19] Guardian staff. Tory leadership election: the full results. The Guardian. 2019.

[20] Council of the EU and the European Council. Timeline - The EU-UK withdrawal agreement. 2024.

[21] Hanretty C. Areal interpolation and the UK’s referendum on EU membership. J Elect Public Opin Parties. 2017;27(4):466–83. doi: 10.1080/17457289.2017.1287081

[22] Alexandre-Collier A. David Cameron, Boris Johnson and the ‘populist hypothesis’ in the British Conservative Party. Comp Eur Polit. 2022;20(5):527–43. doi: 10.1057/s41295-022-00294-5

[23] Evans G, De Geus R, Green J. Boris Johnson to the rescue? How the conservatives won the radical-right vote in the 2019 general election. Polit Stud. 2021. doi: 10.1177/00323217211051191

[24] Alexiadou D. Boris Johnson never took full control of the Tory party – uniting it now seems impossible. The Conversation. 2022.

[25] Usherwood S. Shooting the fox? UKIP’s populism in the post-Brexit era. West Eur Polit. 2019;42(6):1209–29. doi: 10.1080/01402382.2019.1596692

[26] Webb P, Bale T. Shopping for a better deal? Party switching among grassroots members in Britain. J Elect Public Opin Parties. 2021:1–11.

[27] Walker P, Halliday J. Revealed: Ukip membership surge shifts party to far right. The Guardian. 2019.

[28] Heath O, Goodwin M. The 2017 general election, Brexit and the return to two-party politics: An aggregate-level analysis of the result. Polit Q. 2017;88(3):345–58. doi: 10.1111/1467-923x.12405

[29] Cutts D, Goodwin M, Heath O, Surridge P. Brexit, the 2019 general election and the realignment of British politics. Polit Q. 2020;91(1):7–23. doi: 10.1111/1467-923x.12815

[30] Cutts D, Goodwin M, Heath O, Milazzo C. Resurgent remain and a rebooted revolt on the right: Exploring the 2019 European parliament elections in the United Kingdom. Polit Q. 2019;90(3):496–514. doi: 10.1111/1467-923x.12736

[31] Deakin S, Lele P, Siems M. The evolution of labour law: Calibrating and comparing regulatory regimes. Int Labour Rev. 2007;146(3–4):133–62. doi: 10.1111/j.1564-913x.2007.00011.x

[32] Vaubel R. The political economy of labor market regulation by the European Union. Rev Int Organ. 2008;3(4):435–65. doi: 10.1007/s11558-008-9041-6

